# The Median Effective Concentration of Sevoflurane for I‐Gel Laryngeal Mask Insertion in Unpremedicated Children Aged 1–10 Years: A Prospective Concentration‐Finding Study

**DOI:** 10.1002/pdi3.70039

**Published:** 2026-03-29

**Authors:** Zhengwei Gan, Shangyingying Li, Shun Yang, Ling Liu, Qianyu Deng, Yaqiong Tian, Li Yang, Fei Yang, Shengfen Tu

**Affiliations:** ^1^ Department of Anesthesiology, Children’s Hospital of Chongqing Medical University, Ministry of Education Key Laboratory of Child Development and Disorders, Chongqing Key Laboratory of Child Neurodevelopment and Cognitive Disorders National Clinical Research Center for Child Health and Disorders Chongqing China

**Keywords:** children, I‐gel, median effective concentration, sevoflurane

## Abstract

The minimum alveolar concentration of sevoflurane varies with age in children, and the median effective concentration (EC_50_) of sevoflurane for I‐gel insertion in children of different ages has not been reported. The aim of this study was to determine the EC_50_ of end‐tidal sevoflurane maintained for 2.5 min for I‐gel insertion in children aged 1–10 years and also to estimate the 95% effective concentration (EC_95_)_._ This study aimed to recruit children who were scheduled to undergo laparoscopic high ligation of the inguinal hernia sac or laparoscopic high ligature of the sheath process. Children were stratified into three age groups. We employed Dixon's up‐and‐down method in this study. The target end‐tidal sevoflurane concentration was then maintained at 2% for 2.5 min for the first child. The concentration for the subsequent patient was determined based on the response of the previous patient, with adjustments of 0.2%. This study was terminated when seven crossover points were reached. In the 1–3‐year‐old group including 3‐year‐old, EC_50_ was 1.75% (95% confidence interval [CI], 1.72%–2.03%), and EC_95_ was 2.17% (95% CI, 1.96%–2.18%). In the 3–6‐year‐old group including 6‐year‐old, EC_50_ was 1.60% (95% CI, 1.35%–1.83%), and EC_95_ was 1.96% (95% CI, 1.77%–1.98%). In the 6–10‐year‐old group, EC_50_ was 0.96% (95% CI, 0.93%–2.20%) and EC_95_ was 2.36% (95% CI, 2.15%–2.38%). Our study determined the EC_50_ of end‐tidal sevoflurane required for I‐gel insertion in unpremedicated children aged 1–10 years and the results demonstrate to be both safe and effective for pediatric patients.

## 1 Introduction

As a novel supraglottic airway device, the I‐gel laryngeal mask can enhance safety during anesthesia due to its user‐friendly operation, secure attachment to the supraglottic structures, soft elastomer gel material, absence of an inflatable cuff, which helps prevent compression trauma, and its separate gastric channel design, making it widely used for ventilation during anesthesia in pediatric patients [1, 2, 3, 4].

Sevoflurane inhalation induction is frequently employed in pediatric anesthesia, particularly in patients who require maintaining spontaneous ventilation. It is well established that the minimum alveolar concentration (MAC) of sevoflurane varies with age [5]; age is an important determinant of the pharmacokinetic profile of inhaled anesthetics [6]. Kumar et al. divided children aged 1–10 years into small homogeneous groups—Group 1: children aged 1–3 years, Group 2: children aged > 3–7 years, and Group 3: children aged > 7–10 years—to avoid the effect of physiological differences at different ages on sevoflurane concentration [7]. It may be due to the difference in minute ventilation at different ages [8]. A study reported that the median effective concentration (EC_50_) of I‐gel insertion in unpremedicated children aged 1.5–8 years is 0.94% (0.83%–1.06%) during sevoflurane inhalation induction. A comparison between I‐gel and classic laryngeal mask airway (CLMA) was conducted, and the results indicated that the EC_50_ for I‐gel was approximately half of the sevoflurane concentration of CLMA [9]. This suggests that the EC_50_ for I‐gel is lower, but the age range is a bit wide. However, to date, no published studies have reported the EC_50_ of sevoflurane for I‐gel insertion across various pediatric age groups.

Hence, we planned this concentration determination study to determine the EC_50_ of sevoflurane in children aged 1–10 years and divided the research subjects into three age groups according to previous research and the physiological growth characteristics of children [7]: the 1–3‐year‐old group including 3‐year‐old, the 3–6‐year‐old group including 6‐year‐old, and 6–10‐year‐old group. The EC_50_ may serve as an initial concentration for future, more precise trials. Additionally, we estimated the 95% effective concentration (EC_95_) to provide a reference for clinical applications.

## 2 Methods

### 2.1 Ethics

Ethical approval for this study (Approval Number: 2022 [No. 514]) was provided by the Institutional Review Board of Children's Hospital of Chongqing Medical University, Chongqing, China (Chairperson Prof. Zhongyi Lu), on 25 December 2022, and written informed consent was obtained from all subjects' family. The trial was registered prior to patient enrollment at clinicaltrials.gov (ChiCTR2300067869, principal investigator: Shengfen Tu, date of registration: 30/01/2023).

### 2.2 Participants

We prospectively recruited children aged 1–10 years who were scheduled to undergo laparoscopic high ligation of the inguinal hernia sac or laparoscopic high ligature of the sheath process from March 30, 2023, to August 11, 2023, at Children's Hospital of Chongqing Medical University. The American Society of Anesthesiologists (ASA) physical status classification was required to be I or II to be eligible. Exclusion criteria were as follows: upper respiratory tract infections within the preceding 2 weeks, premedication, congenital heart disease, asthma, abnormal liver and kidney function, central nervous system disorders, muscle diseases, inherited metabolic diseases, difficult airways, obesity, or refusal by the child’s family.

### 2.3 Anesthesia Procedures and Study Method

All children were in a fasting status (at least 6 h for formula milk, at least 8 h for solids, and at least 2 h for clear fluids) before entering the operating room. No premedication was administered to any child. The children’s vital signs were monitored, including electrocardiogram (ECG), heart rate (HR), oxygen saturation (SpO_2_), respiratory rate (RR), body temperature (T), and mean arterial pressure (MAP). All children inhaled 8% sevoflurane at a flow rate of 6 L/min with 100% oxygen via a face mask for anesthesia induction, breathing spontaneously. Once the loss of the eyelash reflex was observed, we adjusted the end‐tidal sevoflurane concentration (ETsev) to the predetermined target using the anesthesia workstation (Mindray A9C, China). We then ventilated using the anesthesia machine, ensuring the mask was tightly sealed with both hands. The ETsev and end‐tidal CO_2_ (ETCO_2_) were monitored and recorded.

The target ETsev was maintained for 2.5 min based on our pilot experiments and previous studies [10, 11] before the I‐gel was inserted. The I‐gel (Intersurgical Ltd) sizes were 1.0 (2–5 kg), 1.5 (5–12 kg), 2 (10–25 kg), and 2.5 (25–35 kg). The ETsev was initially set at 2% for the first child. The concentration for the subsequent patient was adjusted based on the response of the previous patient with a decrease or increase of 0.2% using Dixon's up‐and‐down approach [12, 13]. If the insertion failed, the inhalation concentration of sevoflurane was increased or 2 mg/kg propofol was administered intravenously, and the concentration of sevoflurane was increased by 0.2% for the next patient. If the insertion was successful, the concentration was decreased by 0.2% for the next patient. The study ended when 7 crossover points (from failure to success) were collected. After I‐gel insertion, we connected the patient to the anesthesia machine for mechanical ventilation.

We set the mechanical ventilation mode to pressure controlled ventilation‐volume guaranteed (PCV‐VG), the oxygen concentration 35%–40% from an air–oxygen mixture, the gas flow rate 2 L/min, and the tidal volume 8–10 mL/kg. The respiratory rate was adjusted according to the child’s age, the airway peak pressure was maintained below 30 cm H_2_O, and ETCO_2_ was maintained between 35 to 45 mmHg. All patients were administered sufentanil 1 μg/kg, cisatracurium besilate 0.1 mg/kg, and penehyclidine hydrochloride 0.01 mg/kg to deepen anesthesia and meet surgical requirements. Intravenous continuous infusion of propofol 4–6 mg/kg/h, remifentanil 0.2 μg/kg/min, and inhaled 1%–2% sevoflurane were used for anesthesia maintenance until the end of the operation.

### 2.4 Data Acquisition

The HR and the MAP before I‐gel insertion were taken as the baseline values. The criteria for successful insertion required meeting both of the following requirements simultaneously: (1) No movement occurred during I‐gel insertion and within 1 minute after insertion, with “movement” being associated with insertion stimulation, including body and limb movements, teeth clenching, coughing, breath holding, and laryngospasm. (2) HR and MAP changes were less than 20% of the baseline values during I‐gel insertion and within 1 minute after insertion. I‐gel insertion was considered failed if either of the following two conditions occurred: (1) Movement occurred during I‐gel insertion and within 1 minute after insertion. (2) HR or MAP was increased by more than 20% of the baseline values during I‐gel insertion and within 1 minute after insertion.

All these responses were assessed by an observer who was unaware of the ETsev concentration, and all I‐gel insertions were performed by a single anesthesiologist to ensure a smooth process. We recorded the patients’ general information, including age, sex, weight, whether they were crying or quiet, and ASA physical status. Vital signs at various time points and adverse events were recorded, including HR, MAP, laryngospasm, and tracheospasm.

### 2.5 Statistical Analysis

The EC_50_ and EC_95_ of end‐tidal sevoflurane were statistically calculated using isotonic regression with Dixon's up‐and‐down sequential allocation method. We used the bootstrap algorithm with 2000 replicates to acquire the 95% confidence intervals (CI) for EC_50_ and EC_95_. For higher accuracy, we used the estimate μ3 to represent the effective concentration at which the target effect (0.5) was achieved [14]. The statistical analysis was performed using the R 4.2.0 software package (R Foundation for Statistical Computing, Vienna, Austria). Continuous variables of patients’ characteristics and information were expressed as the mean ± standard deviation and analyzed by the *t* test; categorical variables were expressed as frequency and/or percentage and analyzed by Fisher’s exact test, using SPSS for Windows version 22.0 (SPSS Inc., Chicago, IL, USA), with *p* < 0.05 considered statistically significant.

## 3 Results

**FIGURE 1:**
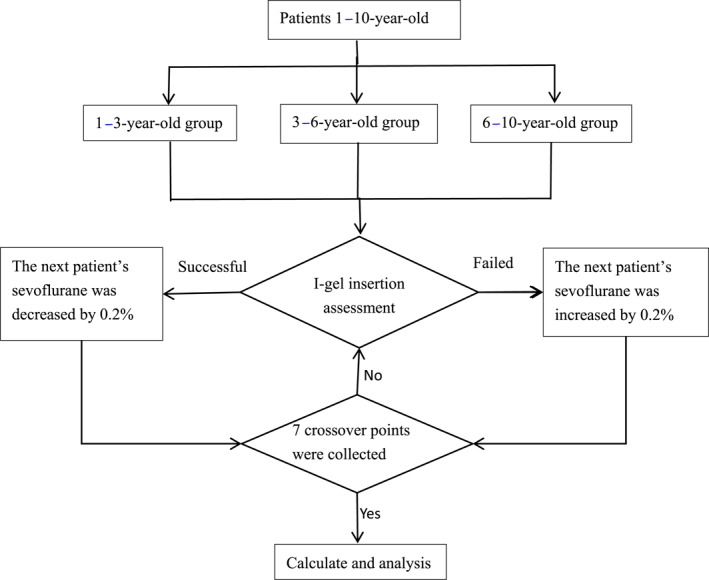
Flow diagram of the study.

A total of 80 children undergoing laparoscopic high ligation of the inguinal hernia sac or laparoscopic high ligature of the sheath process were enrolled in this prospective study; a flowchart depicting patient recruitment is presented in Figure 1.

There were 10 patients with failure in the 1–3‐year‐old group, 12 in the 3–6‐year‐old group, and 13 in the 6–10‐year‐old group. Among the patients with failure in the 1–3‐year‐old group, only two had an HR increase greater than 20% above baseline values during I‐gel insertion; no body or limb movement occurred, and no reinsertion of the I‐gel was necessary. “Movement” occurred during I‐gel insertion for all other patients with failure, accompanied by changes in vital signs.

Among them, one patient in the 3–6‐year‐old group clenched the laryngeal mask with their teeth, and two patients in the 6–10‐year‐old group experienced the same issue. We increased the inhalation concentration of sevoflurane or administered 2 mg/kg propofol intravenously to patients whose insertion failed.

If the I‐gel was properly positioned and ventilated satisfactorily, it was then directly connected to the anesthesia machine for mechanical ventilation. If not, the I‐gel needed to be reinserted. There were 8 patients with failure requiring reinsertion of the I‐gel in the 1–3‐year‐old group, 12 in the 3–6‐year‐old group, and 13 in the 6–10‐year‐old group. All patients completed the operation with stable vital signs and without any adverse events.

Patients’ sex, age (in months), weight (in kg), whether they were crying or quiet, time to loss of the eyelash reflex, time to target ETsev, and other variables for the three groups are presented in Table 1. Details for each group are provided in Tables 2, 3, 4. There were no significant differences in age, weight, time to loss of the eyelash reflex, time to target ETsev, crying, and other variables between cases without and with failure within each group.

**TABLE 1:** Patients' characteristics and information.

Variables	1–3 years old (*n* = 22)	3–6 years old (*n* = 26)	6–10 years old (*n* = 32)
Sex (M/F, *n*)	19/3	19/7	21/11
Age (months)	24.9 ± 7.0	51.9 ± 10.7	85.3 ± 11.7
Weight (kg)	12.0 ± 1.6	17.2 ± 2.7	24.1 ± 4.9
Crying/quiet (*n*)	20/2	11/15	3/29
Incidence of crying (%)	90.9	42.3	9.4
Time to loss of the eyelash reflex (s)	64.1 ± 22.1	68.9 ± 23.5	71.6 ± 16.3
Time to target concentration (s)	148.0 ± 52.1	153.4 ± 65.4	186.0 ± 76.7
Success/failure (*n*)	12/10	14/12	19/13
Success rate (%)	54.5	53.8	59.4
EC_50_ (95% CI) of ET_sev_ (%)	1.75 (1.72–2.03)	1.60 (1.35–1.83)	0.96 (0.93–2.20)
EC_95_ (95% CI) of ET_sev_ (%)	2.17 (1.96–2.18)	1.96 (1.77–1.98)	2.36 (2.15–2.38)
Adverse events	No	No	No

*Note:* Data are presented as the number of patients or mean ± standard deviation, unless otherwise specified.

Abbreviations: EC_50_, median effective concentration; EC_95_, 95% effective concentration; ETsev, end‐tidal sevoflurane concentration.

**TABLE 2:** Characteristics of patients with success and failure in the 1–3‐year‐old group.

Variables	Success (*n* = 12)	Failure (*n* = 10)
Sex (M/F, *n*)	10/2	9/1
Age (months)*	22.7 ± 7.3	27.6 ± 5.7
Weight (kg)*	11.1 ± 1.3	13.2 ± 1.1
Crying/quiet (*n*)*	12/0	8/2
Incidence of crying (%)	100	80
Time to loss of the eyelash reflex (s)*	66.7 ± 25.3	61.0 ± 18.4
Time to target concentration (s)*	164.3 ± 53.4	128.4 ± 45.5
Coughing	/	None
Breath holding	/	None
Clenching of teeth	/	None
Laryngospasm	/	None
Body and limb movements	/	8
Only vital signs changed by > 20% of the baseline values (HR or MAP)	/	2 (HR)
Vital signs at baseline value (sedate)		
HR (bpm/min)*	100.8 ± 14.6	100.3 ± 15.8
MAP (mmHg)*	56.9 ± 7.7	63.8 ± 12.1
Number of I‐gel insertions	1	1 or 2

*Note:* Data are presented as the number of patients or mean ± standard deviation, unless otherwise specified.

Abbreviations: HR, heart rate; MAP, mean arterial pressure.

^*^ *p* > 0.05.

**TABLE 3:** Characteristics of patients with success and failure in the 3–6‐year‐old group.

Variables	Success (*n* = 14)	Failure (*n* = 12)
Sex (M/F, *n*)	11/3	8/4
Age (months)*	51.3 ± 10.9	52.6 ± 10.8
Weight (kg)*	17.5 ± 2.7	16.8 ± 2.8
Crying/quiet (*n*)*	6/8	5/7
Incidence of crying (%)	42.9	41.7
Time to loss of the eyelash reflex (s)*	69.6 ± 23.2	68.0 ± 24.8
Time to target concentration (s)*	154.3 ± 57.7	152.4 ± 76.2
Coughing	/	None
Breath holding	/	None
Clenching of teeth	/	1
Laryngospasm	/	None
Body and limb movements	/	11
Only vital signs changed by > 20% of the baseline values (HR or MAP)	/	None
Vital signs at baseline value (sedate)		
HR (bpm/min)*	81.9 ± 8.8	85.9 ± 14.5
MAP (mmHg)*	58.6 ± 7.0	59.9 ± 7.4
Numbers of I‐gel insertions	1	2

*Note:* Data are presented as the number of patients or mean ± standard deviation, unless otherwise specified.

Abbreviations: HR, heart rate; MAP, mean arterial pressure.

^*^ *p* > 0.05.

**TABLE 4:** Characteristics of patients with success and failure in the 6–10‐year‐old group.

Variables	Success (*n* = 19)	Failure (*n* = 13)
Sex (M/F, *n*)	14/5	7/6
Age (months)*	83.4 ± 9.2	88.1 ± 14.5
Weight (kg)*	23.1 ± 4.0	25.6 ± 5.8
Crying/quiet (*n*)*	2/17	1/12
Incidence of crying (%)	10.5	7.7
Time to loss of the eyelash reflex (s)*	74.5 ± 15.4	67.4 ± 17.3
Time to target concentration (s)*	185.4 ± 76.9	186.8 ± 79.6
Coughing	/	None
Breath holding	/	None
Clenching of teeth	/	2
Laryngospasm	/	None
Body and limb movements	/	11
Only vital signs changed by > 20% of the baseline values (HR or MAP)	/	None
Vital signs at baseline value (sedate)		
HR (bpm/min)*	79.7 ± 10.7	81.4 ± 17.8
MAP (mmHg)*	61.4 ± 4.2	63.5 ± 11.1
Number of I‐gel insertions	1	2

*Note:* Data are presented as the number of patients, mean ± SD.

Abbreviations: HR, heart rate; MAP, mean arterial pressure.

^*^ *p* > 0.05.

**FIGURE 2:**
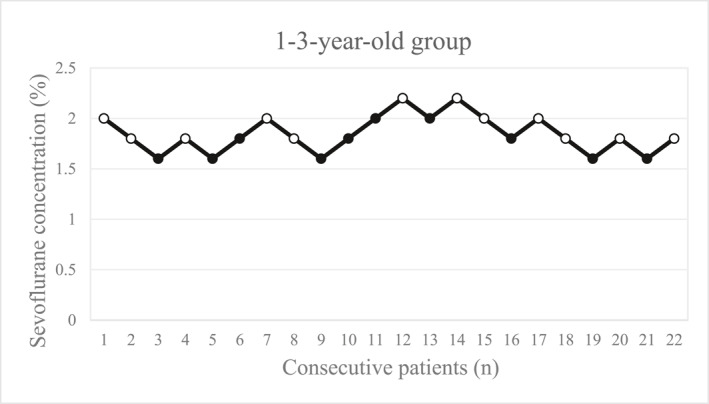
The concentration of sevoflurane in patients aged 1–3 years (*n* = 22). Each solid circle represents a failed I‐gel insertion (movement, difficult mouth opening, clenching of teeth, coughing, breath holding, laryngospasm), and each open circle represents a successful I‐gel insertion.

**FIGURE 3:**
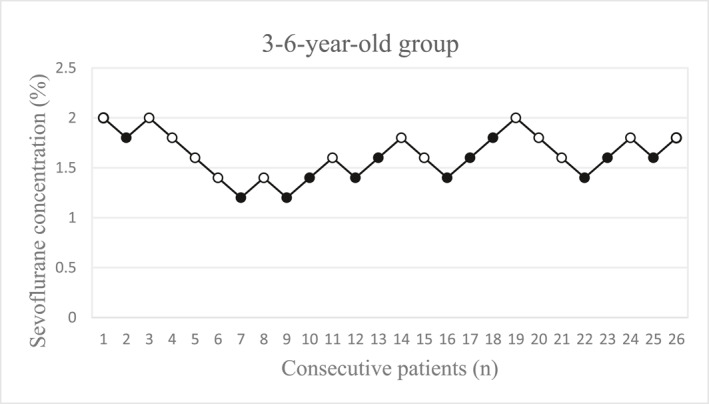
The concentration of sevoflurane in patients aged 3–6 years (*n* = 26). Each solid circle represents a failed I‐gel insertion (movement, difficult mouth opening, clenching of teeth, coughing, breath holding, laryngospasm), and each open circle represents a successful I‐gel insertion.

**FIGURE 4:**
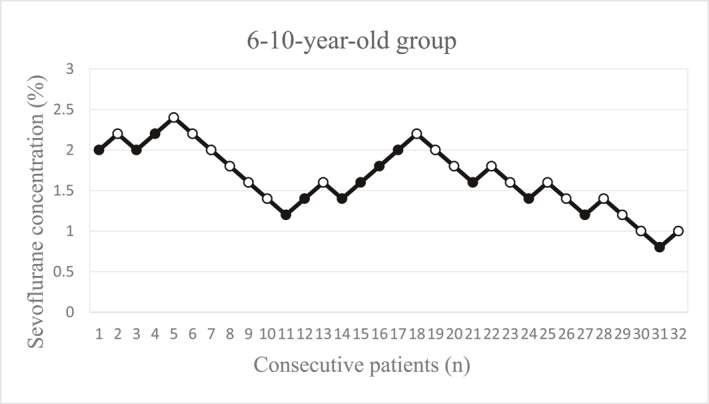
The concentration of sevoflurane in patients aged 6–10 years (*n* = 32). Each solid circle represents a failed I‐gel insertion (movement, difficult mouth opening, clenching of teeth, coughing, breath holding, laryngospasm), and each open circle represents a successful I‐gel insertion.

The sequences of patients with success (open circles) and those with failure (solid circles) are shown in Figures 2, 3, 4 for the different groups.

The EC_50_ and EC_95_ of end‐tidal sevoflurane for I‐gel laryngeal mask insertion were estimated to be 1.75% (95% CI, 1.72%–2.03%) and 2.17% (95% CI, 1.96%–2.18%) in the 1–3‐year‐old group, 1.60% (95% CI, 1.35%–1.83%) and 1.96% (95% CI, 1.77%–1.98%) in the 3–6‐year‐old group, and 0.96% (95% CI, 0.93%–2.20%) and 2.36% (95% CI, 2.15%–2.38%) in the 6–10‐year‐old group. No adverse events occurred during intraoperative or postoperative visits.

## 4 Discussion

Our study affirms that I‐gel is a safe airway device for pediatric patients, characterized by a short insertion time, a high success rate for insertion, and a low EC_50_ of sevoflurane required for I‐gel insertion. This aligns with previous research indicating that the I‐gel can secure the airway in short‐duration surgical procedures without the need for endotracheal intubation [15]. Additionally, the I‐gel serves as a valuable rescue device for managing patients with difficult airways during emergencies, such as those with difficult mask ventilation or failed intubation [16, 17]. Sevoflurane inhalational induction is a prevalent method for anesthesia induction in pediatric clinical practice [18], as it allows for spontaneous breathing and facilitates the placement of intravenous access. However, concerns have been raised regarding the adverse events associated with sevoflurane, particularly when high concentrations are used for induction [19, 20]. Determining the EC_50_ can help reduce the dose of sevoflurane while ensuring safety and effectiveness in clinical practice. This approach aligns with the goal of using the lowest effective anesthetic concentration to mitigate potential side effects, such as hemodynamic instability and respiratory depression. A recent study has reported that the EC_50_ of I‐gel insertion in unpremedicated children aged 1.5–8 years during sevoflurane inhalation induction is 0.94% (CI, 0.83%–1.06%) [9], which was different from the findings of our study. This discrepancy may be attributed to pharmacological differences across various age groups, as well as variations in the maintained time of ETsev; the study in question maintained ETsev for 8–10 min, whereas our study utilized a 2.5‐min maintenance period.

It is well established that the MAC of inhalation anesthetics decreases with increasing age, as demonstrated in previous studies [21, 22, 23]. This age‐related decrease in MAC may be associated with several physiological changes in the central nervous system, including decreased cell density, reduced cerebral oxygen consumption, and decreased cerebral blood flow [24, 25]. Kumar et al. conducted a study evaluating the optimal time for intravenous (IV) cannulation after sevoflurane induction in children across different age groups. They observed that children aged 1–3 years, induced with 8% sevoflurane, had a significantly shorter mean time for IV cannulation (54 s) compared to those over 3 years of age, with the older groups having times of 105 and 144 s. This finding suggests that younger children achieve a higher MAC of sevoflurane more rapidly than their older counterparts [7]. In our study, we enrolled children aged 1–10 years and stratified them into three age cohorts based on physiological growth characteristics and findings from prior research: 1–3‐year‐old, 3–6‐year‐old, and 6–10‐year‐old. We determined the EC_50_ of sevoflurane required for successful I‐gel insertion within each age cohort. Our results revealed a decrease in EC_50_ with increasing age across all three cohorts, a pattern consistent with previous studies.

In the majority of studies, the target ETsev is maintained for 10–15 min to achieve blood–brain partial pressure equilibrium for sevoflurane [26, 27]. In clinical practice, it is recognized that maintaining the target ETsev for an extended period may not be practical, especially in busy operating rooms. A study by Sethi et al. specifically addressed this concern by determining the EC_50_ and EC_95_ for CLMA insertion at equilibration times of 2.5 and 5 min in children aged 2–8 years. They found that the EC50 was 1.1% (CI, 0.9–1.2) for 2.5 min and 1.2% (0.6–1.8) for 5 min [10]. In a study by Kennedy et al., it was determined that the mean time for laryngeal mask airway insertion after inhaling sevoflurane was significantly shorter for women, with a mean time of 2.25 min [11]. In our study, we set the maintenance time to 2.5 min, similar to the equilibration time point established in previous research. Through this methodology, we calculated the EC_50_ and estimated the EC_95_ for I‐gel insertion across different age groups. It is important to note that these results are indicative of the concentration at the specific equilibration time of 2.5 min and may not reflect the concentrations required at other time points.

We must acknowledge several limitations inherent in our study. Primarily, the sample was restricted to unpremedicated children, who exhibited agitation upon entering the operating room, particularly younger children, who were often in tears. This emotional distress could potentially impact lung capacity, thereby influencing the outcomes of our trial. Sedative premedication is instrumental in mitigating anxiety and reducing the emotional distress experienced by children, particularly in the context of preoperative settings. Commonly employed sedatives, such as midazolam and dexmedetomidine, have been widely used in pediatric anesthesia in recent years [28]. Research has demonstrated that premedication can effectively lower the MAC of sevoflurane required for laryngeal mask airway insertion [27, 29]. Specifically, oral midazolam and intranasal dexmedetomidine have been shown to significantly decrease the sevoflurane EC_50_ for this procedure by 17% and 21%, respectively [27]. Our findings may not be generalizable to children who have received premedication. Consequently, future trials should assess the impact of premedication across various age groups. Additionally, we recorded baseline vital signs after sevoflurane inhalation induction for all participants. This approach was necessary because some unpremedicated patients exhibited agitation and crying prior to anesthesia, which can significantly influence heart rate and blood pressure measurements. Thirdly, the EC_95_ values were extrapolated from the EC_50_, a method that may be less precise given the small sample sizes in our study. Specifically, the EC_95_ values were 2.17% for the infant group, 1.96% for the preschool group, and 2.36% for the school‐age group. For anesthesiologists, higher percentile effective doses, such as EC_95_ or EC_99_, are more desirable, as they offer greater clinical applicability and guidance [13, 30]. In subsequent studies, the EC_50_ values identified in our research can serve as a preliminary concentration for additional trials aimed at directly determining the EC_95_. Lastly, there is a dearth of high‐quality literature regarding the duration for which the target ETsev should be maintained prior to I‐gel insertion. Future experiments could investigate the optimal maintenance durations for children across different age groups.

## 5 Conclusions

This study determined the EC_50_ of end‐tidal sevoflurane required for I‐gel insertion in unpremedicated children aged 1–10 years. The identified EC_50_ can serve as an initial concentration for subsequent research. Furthermore, this method of anesthesia induction has been demonstrated to be both safe and effective for pediatric patients.

## Author Contributions

Zhengwei Gan and Shangyingying Li helped design and perform the study, and contributed significantly to the analysis and manuscript preparation. Shengfen Tu conceived, designed and funded the study. Qianyu Deng, Yaqiong Tian, Li Yang and Fei Yang performed the quality assessment. Shun Yang and Ling Liu helped to perform statistical analyses and search strategies. Zhengwei Gan and Shangyingying Li drafted and helped to perform the study. All authors have read and approved the manuscript.

## Funding

This work was supported by the National Clinical Research Center for Child Health and Disorders General Program of Clinical Medical Research (NCRCCHD‐2022‐GP‐0X) and the Natural Science Foundation of Chongqing (CSTB2023NSCQ‐MSX0434).

## Conflicts of Interest

The authors declare no conflicts of interest.

## Data Availability

The data that support the findings of this study are available on request from the corresponding author. The data are not publicly available due to privacy and ethical restrictions.
